# Identification of a Male-Produced Pheromone Component of the Citrus Longhorned Beetle, *Anoplophora chinensis*

**DOI:** 10.1371/journal.pone.0134358

**Published:** 2015-08-04

**Authors:** Laura Hansen, Tian Xu, Jacob Wickham, Yi Chen, Dejun Hao, Lawrence M. Hanks, Jocelyn G. Millar, Stephen A. Teale

**Affiliations:** 1 Department of Environmental and Forest Biology, College of Environmental Science and Forestry, State University of New York, Syracuse, NY, 13210, United States of America; 2 Key Lab of Analytical Chemistry for Living Biosystems, Institute of Chemistry, Chinese Academy of Sciences, Beijing, China; 3 Department of Forest Protection, Nanjing Forestry University, Nanjing, China; 4 Department of Entomology, University of Illinois, Urbana-Champaign, IL, 61801, United States of America; 5 Departments of Entomology and Chemistry, University of California Riverside, Riverside, CA, 92521, United States of America; United States Department of Agriculture, Beltsville Agricultural Research Center, UNITED STATES

## Abstract

The Asian wood-boring beetle *Anoplophora chinensis* (Forster) (Coleoptera: Cerambycidae) is an important pest of hardwood trees in its native range, and has serious potential to invade other areas of the world through worldwide commerce in woody plants and wood products. This species already has been intercepted in North America, and is the subject of ongoing eradication efforts in several countries in Europe. Attractants such as pheromones would be immediately useful as baits in traps for its detection. Because long-range pheromones are frequently conserved among closely related species of cerambycids, we evaluated two components of the volatile pheromone produced by males of the congener *A*. *glabripennis* (Motschulsky), 4-(*n*-heptyloxy)butan-1-ol and 4-(*n*-heptyloxy)butanal, as potential pheromones of *A*. *chinensis*. Both compounds subsequently were detected in headspace volatiles from male *A*. *chinensis*, but not in volatiles from females. Only 4-(*n*-heptyloxy)butanol elicited responses from beetle antennae in coupled gas chromatography-electroantennogram analyses, and this compound attracted adult *A*. *chinensis* of both sexes in field bioassays. These data suggest that 4-(*n*-heptyloxy)butan-1-ol is an important component of the male-produced attractant pheromone of *A*. *chinensis*, which should find immediate use in quarantine monitoring for this pest.

## Introduction

The citrus longhorned beetle, *Anoplophora chinensis* (Forster), is a polyphagous wood-boring cerambycid beetle that is native to East Asia [1]. Its broad host range (plant species in at least 36 families) and ability to infest and kill living trees make this species an important pest in its native range, and a potentially devastating invasive species in other parts of the world [2]. The beetle already has become established in several European countries, where it is predicted to cause massive environmental damage and major economic damage if it cannot be eradicated [2–4]. It also has been detected at least twice in North America, in Georgia and Washington State, but appears not to have become established [3].

Current eradication methods for invasive longhorned beetles such as *A*. *chinensis*, and the congeneric Asian longhorned beetle, *A*. *glabripennis* (Motschulsky), rely on visual surveys for physical signs of infestation, such as the characteristic holes in tree trunks and branches left by emerging adults. Once an infested tree is discovered, all potential host trees are removed within an encircling containment zone [2]. These methods are problematical, however, in that it is virtually impossible to detect all emergence holes in tall standing trees by visual surveys alone, and it may be some time before new infestations outside containment zones are detected. Thus, current methods are ineffective in delimiting the true distribution of the pest. These factors hinder eradication efforts, because eradication requires early detection before beetles can disperse and establish new infestations.

Semiochemical-based trapping has the potential to greatly improve the sensitivity, reliability, and efficiency of detecting populations of *A*. *chinensis*, even at low densities in the early stages of establishment. Such traps already are used in monitoring the spread of the invasive cerambycid *Tetropium fuscum* (F.) (Spondylidinae) in Canada [5], and in Europe for mass trapping the cerambycids *Monochamus galloprovincialis* (Olivier) (Lamiinae) [6] and *Hylotrupes bajulus* (L.)(Cerambycinae) [7]. These results indicate that pheromone-baited traps can constitute efficient and reliable tools for monitoring and managing cerambycids [8,9], and semiochemically-baited traps currently are being developed to specifically target *A*. *glabripennis* [10].

Because of the economic importance of *A*. *chinensis* in its native range in Asia, there has been considerable research on its chemical ecology. Thus, it has been shown that a complex blend of cuticular lipids on the cuticle of females serves as a mate recognition signal for males [11–14]. Although it has been reported that females are attracted by odors from freshly-killed male beetles [15], longer-range pheromones have yet to be identified. Research over the past decade, however, has revealed that pheromone components of cerambycids often are conserved among closely-related, and even among more distantly-related species, with the same chemical serving as the sole or dominant pheromone component of multiple species. For example, within the tribe of *A*. *chinensis* (Monochamini, or Lamiini of some authors), 2-(undecyloxy)ethanol (monochamol) is the male-produced pheromone, or the likely pheromone for at least six species of *Monochamus* [16], as well as species in other genera [17]. Furthermore, a close structural analog of monochamol, 4-(n-heptyloxy)butan-1-ol, is produced by male *A*. *glabripennis*, and believed to be an important component of its volatile pheromone [18,19]. Similarly, (*E*)-6,10-dimethylundecadien-2-ol (fuscumol), and the corresponding acetate and ketone are known or suspected male-produced pheromone components for numerous species in the cerambycid subfamilies Lamiinae and Spondylidinae [20].

Taken together, this earlier research suggested that the pheromone of *A*. *chinensis* is likely to be produced by males, with a reasonable probability that it has the same or a very similar hydroxyether structure to those of closely related species such as *A*. *glabripennis*. This hypothesis was supported by preliminary field bioassays conducted in China (see Supporting Information) which suggested that adult *A*. *chinensis* were significantly attracted to traps baited with 4-(*n*-heptyloxy)butan-1-ol, alone or blended with other chemicals. Here, we confirm that 4-(*n*-heptyloxy)butan-1-ol is an important pheromone component for *A*. *chinensis* by: 1) demonstrating that 4-(*n*-heptyloxy)butan-1-ol and the corresponding aldehyde are produced sex-specifically by males, 2) demonstrating that antennae of both males and females respond strongly to the alcohol, but not to the aldehyde, and 3) confirming with field bioassays that both sexes are significantly attracted to traps baited with 4-(*n*-heptyloxy)butan-1-ol alone, or blended 1:1 with the aldehyde.

## Methods

### Sources of Adult Insects

No official permits were required for any of the three field sites in China. Two of the field sites, the Xiashu Forestry Station and the main campus of Nanjing Forestry University (NFU) are controlled by Nanjing NFU and our cooperator, Dr. Dejun Hao arranged informal permission from NFU for our experiments and collections. The third field site, Nanjing Botanical Garden (NBG), also required no official written permission for our activities there. Unofficial permission was similarly obtained by another cooperator, Dr. Hongjiang Wang, who is the head of the plant protection unit of NBG. Neither the trapping experiments nor the collection of adults involved endangered or protected species. Adult *A*. *chinensis* for identification of volatiles were hand collected at Nanjing Forestry University (32°04'46.95"N 118°48'47.40"E) and Xiashu Forestry Station (32°07'19.38"N 119°12'46.64"E) in Nanjing, China, during May—July 2014. Insects were held at 25–30C under natural light and provided with cuttings of *Salix babylonica* L. and *Platanus x acerifolia* (Aiton) Willd as food. Beetles for coupled gas chromatography-electroantennogram detection studies (GC-EAD) were shipped to the Institute of Chemistry, Chinese Academy of Sciences, Beijing.

### Collection of Insect-produced Volatiles

Headspace volatiles were collected for 24 h from single males (N = 9), single females (N = 9), paired males and females (N = 9), or groups of three females (N = 5), held in aeration chambers with twigs of *S*. *babylonica*. Controls consisted of twigs alone (N = 6) and empty chambers (N = 6). Beetles with twigs were placed in stoppered glass 2-l flasks through which air was drawn (30–500 ml/min) with a portable vacuum pump (Airlight, SKC Inc., Eighty Four, PA, USA). Incoming air was purified with a scrubber containing 6–14 mesh charcoal. Volatiles were collected with a trap consisting of 200 mg of the adsorbent Porapak Q (Sigma-Aldrich, Milwaukee, WI, USA) held between glass wool plugs in a glass tube. The Porapak was first cleaned by Soxhlet-extraction with chloroform, then rinsed immediately before use with dichloromethane. Traps were eluted with 0.5 ml of dichloromethane.

### Analysis of Insect-produced Volatiles

In Syracuse, aeration samples were screened for 4-(n-heptyloxy)butan-1-ol and 4-(n-heptyloxy)butanal (henceforth “alcohol” and “aldehyde”) by coupled gas chromatography-mass spectrometry (GC-MS; Agilent 7890A GC interfaced to a 5975 mass selective detector in EI mode, 70 eV; Agilent Technologies, Santa Clara, CA, USA). The GC was fitted with an HP-WAX column (60 m × 0.2 mm ID × 0.25 μm film thickness; Agilent Technologies). Injections were made in splitless mode with an injector temperature of 250C, the oven was programmed from 40C for 1 min, ramped 3C min^-1^ to 220C, hold at 220C for 10 min. The carrier gas was helium at a constant flow rate of 1 ml min^-1^. The two compounds were quantified using linear regression standard curves developed with solutions of synthetic standards of known concentration.

### Coupled Gas-Chromatography-Electroantennogram Detection (GC-EAD)

Responses of the antennae of adult male and female *A*. *chinensis* (N = 3 for each sex) to synthetic standards (100 ng/ul in pentane) of the alcohol and aldehyde, and to natural volatiles of the male and host plant (sample collection as described above) were assessed using an Agilent 7890A GC coupled to an EAD system (Syntech, Kirchzarten, Germany). For analysis of the synthetic standards, the GC was equipped with an HP-5MS capillary column (30 m length x 0.25 mm ID x 0.25 μm film thickness; Agilent Technologies). For the natural extracts, the GC was fitted with a DB-WAX capillary column (30 m length x 0.25 mm ID x 0.25 μm film thickness; Agilent Technologies). Injector temperature was 260C, injection mode was splitless, and the GC oven was programmed from 40C for 1 min, ramped to 280C at 10C min^-1^, hold for 20 min. The carrier gas was nitrogen at a constant flow rate of 1 ml^-1^. The column effluent was divided 1:3 between the FID and EAD detectors with a splitter (Part# G3180 61500 Rev. C, Agilent Technologies) and two transfer lines of equal length (deactivated capillary column; 0.25 mm ID, Alltech Associates, Deerfield, IL), with 30 ml/min nitrogen makeup gas. The branch to the EAD preparation passed through the wall of the GC via a heated (220C) transfer line (TC-02, Syntech) to the antennal preparation. The antennal preparation was positioned inside an odor delivery tube, and column effluent was delivered to the antennal preparation with charcoal-filtered, humidified air (200 ml/min). The EAD was enclosed in a Faraday cage on a separate table adjacent to the GC. Single antennae were severed at three segments above the scape, and mounted on a Syntech electrode with Spectra 360 electrode gel (Parker Laboratories, Fairfield, NJ, USA).

EAD signals were recorded using a Syntech IDAC-4 data acquisition system and GC-EAD Pro software. FID signals were recorded simultaneously using Chemstation software (Agilent Technologies). Unfiltered signals were amplified 2x. A minimum of three consecutive antennal responses were recorded before a compound was scored as eliciting a positive response to a particular test compound.

### Field Bioassays of Candidate Pheromones

Attraction of adult *A*. *chinensis* to the alcohol alone, the aldehyde alone, and a 1:1 blend of the two was tested in field trials at the Nanjing Botanical Garden (32°03'23.05"N 118°49'46.10"E) during 2–29 July 2014 (12 replicates), and the Xiashu Forestry Station during 26 June—21 July 2014 (3 replicates). Pheromone lures were hung in the center of flight-intercept panel traps (IPM Technologies, Portland, OR, USA) that were coated with Fluon (Northern Products, Woonsocket, RI, USA). Pheromone lures were prepared by heat-sealing 25 mg of pheromone diluted to 1 ml with isopropanol in polyethylene tubing (7 cm x 4.9 cm, E.P.C., Los Angeles, CA, USA). Release rates, as measured in the laboratory by aeration on Porapak Q at 34C followed by GC-MS quantification, were 225±77.2 μg/d for the alcohol and 243±112 μg/d for the aldehyde. Control lures contained 1 ml of isopropanol. Pheromone chemicals were synthesized as previously described [6]. Traps were hung in trees 1–2 m above the ground, ~10 m apart. Collection cups contained a 50:50 (v/v) solution of propylene glycol and water to kill and preserve trapped insects. Trapped insects were collected weekly, at which time lures were rotated one position within trap lines. Lures were replaced every 1–2 wk as needed. Neither the trapping experiments nor the collection of adults involved endangered or protected species.

### Statistical Analysis

Differences among treatments in the mean number of male and female *A*. *chinensis* captured per trap and week were tested using Statistica v. 10 [21]. Transformed (log[x+1]) trap catches of males were tested by ANOVA, having met the assumption of homoscedasticity (Shapiro-Wilks’ and Levene’s tests). Means were compared using Duncan’s range test. However, transformation failed to resolve heteroscedacticity in trap capture data for females (due to zeros in the control treatment), and treatment effects therefore were tested with the Kruskal-Wallis nonparametric ANOVA followed by the median test to assess differences among treatments. Field data for the two study sites were combined because preliminary analyses revealed no significant lure × site interactions (ANOVA *F*_3,52_ = 0.33, *P* = 0.81).

## Results

### Analysis of Insect-produced Volatiles

**Fig 1:**
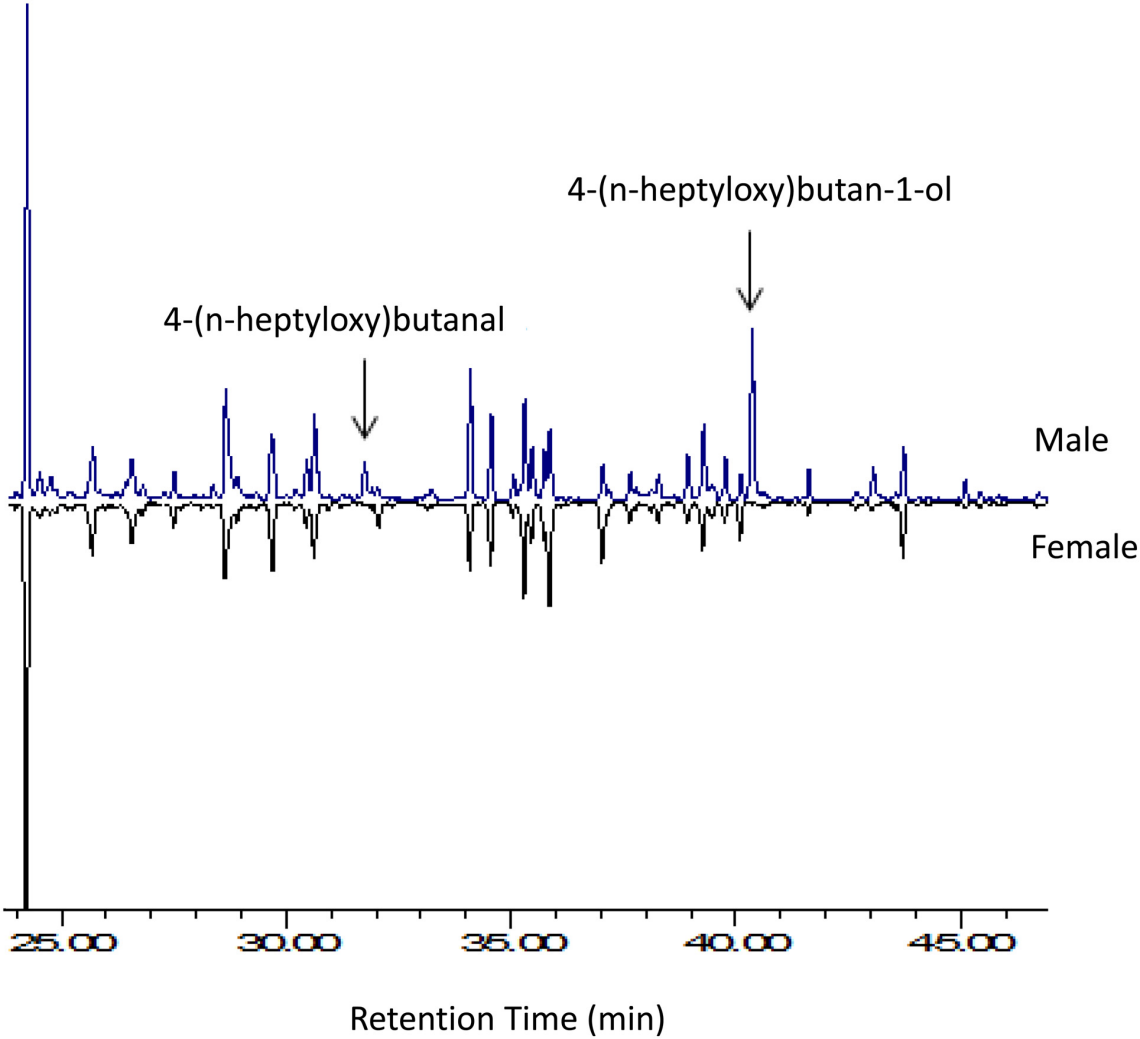
Representative total ion chromatograms of extracts of headspace volatiles from adult male (upper trace) and female *Anoplophora chinensis* (lower, inverted trace) aerated on host plant material. Only males produced detectable quantities of 4-(n-heptyloxy)butan-1-ol and 4-(n-heptyloxy)butanal.

**Fig 2:**
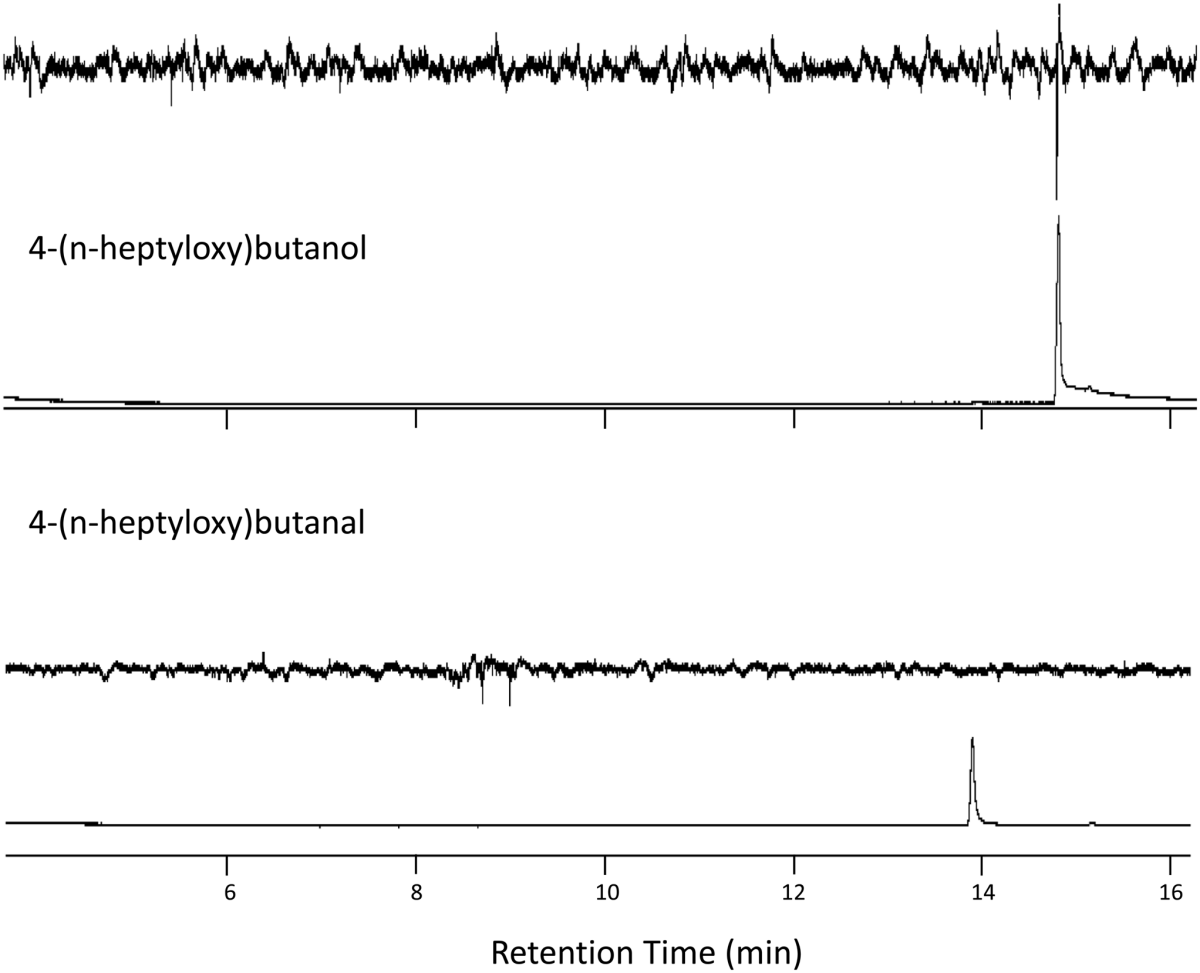
Representative GC-EAD analyses that tested for responses from the antenna of an adult female *Anoplophora chinensis* to synthetic 4-(n-heptyloxy)butanal and 4-(n-heptyloxy)butan-1-ol. Only the latter compound elicited responses from the antennae of both sexes.

**Fig 3:**
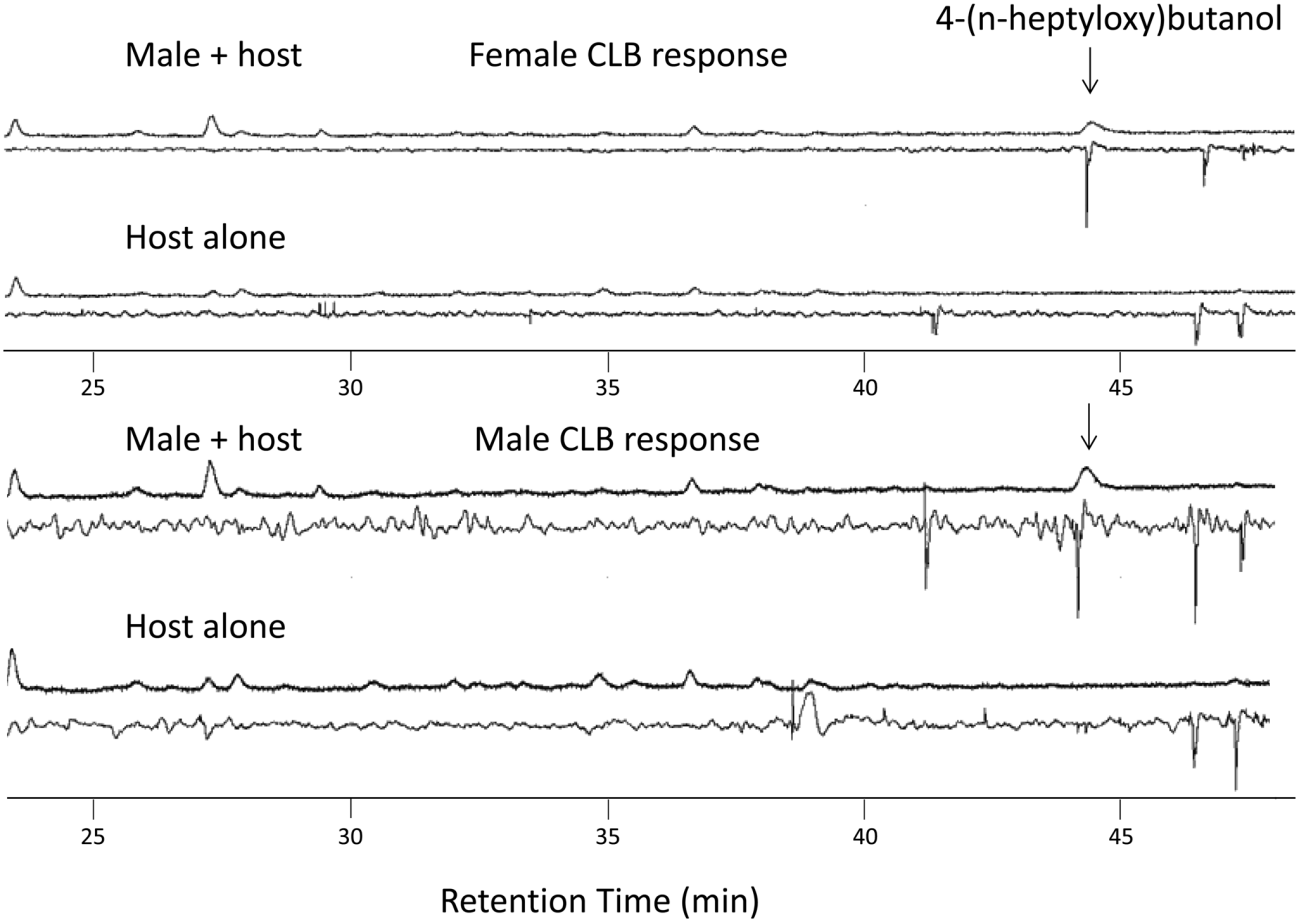
Representative GC-EAD analyses that tested for responses from the antennae of adult female (above) and male (below) *Anoplophora chinensis* to volatiles produced by males and host plants, and host plants alone. The only insect-produced compound to elicit an antennal response was 4-(n-heptyloxy)butan-1-ol.

Extracts of headspace volatiles from male *A*. *chinensis* and from paired males and females consistently contained two compounds (absent in extracts from females, and controls) with retention times and mass spectra matching those of synthetic standards of the aldehyde and the alcohol (Table 1, Fig 1). Extracts of individual males contained both chemicals, whereas those of males paired with females contained only the alcohol (Table 1). No other insect-produced compounds were detected in any of the extracts. Antennae of adult *A*. *chinensis* of both sexes responded strongly to the synthetic and natural alcohol, but not to the aldehyde (Figs 2 and 3). Antennal responses of both males and females did not indicate the presence of active, insect-produced volatiles other than the alcohol but at least two host volatiles elicited responses (Fig 3).

**Table 1:** Chemicals present in extracts from headspace aerations of adult male and female *Anoplophora chinensis*.

Sex		4-(*n*-heptyloxy)butan-1-ol			4-(*n*-heptyloxy)butanal	
	N	No.producing	Quantity (μg/day)	No. producing	Quantity (μg/day)
Single male	9	6	1.01 ± 0.84	4	0.32 ± 0.25
Male female pair	9	4	0.12 ± 0.04	0	0
Single female	9	0	0	0	0

### Field Bioassays of Candidate Pheromones

**Fig 4:**
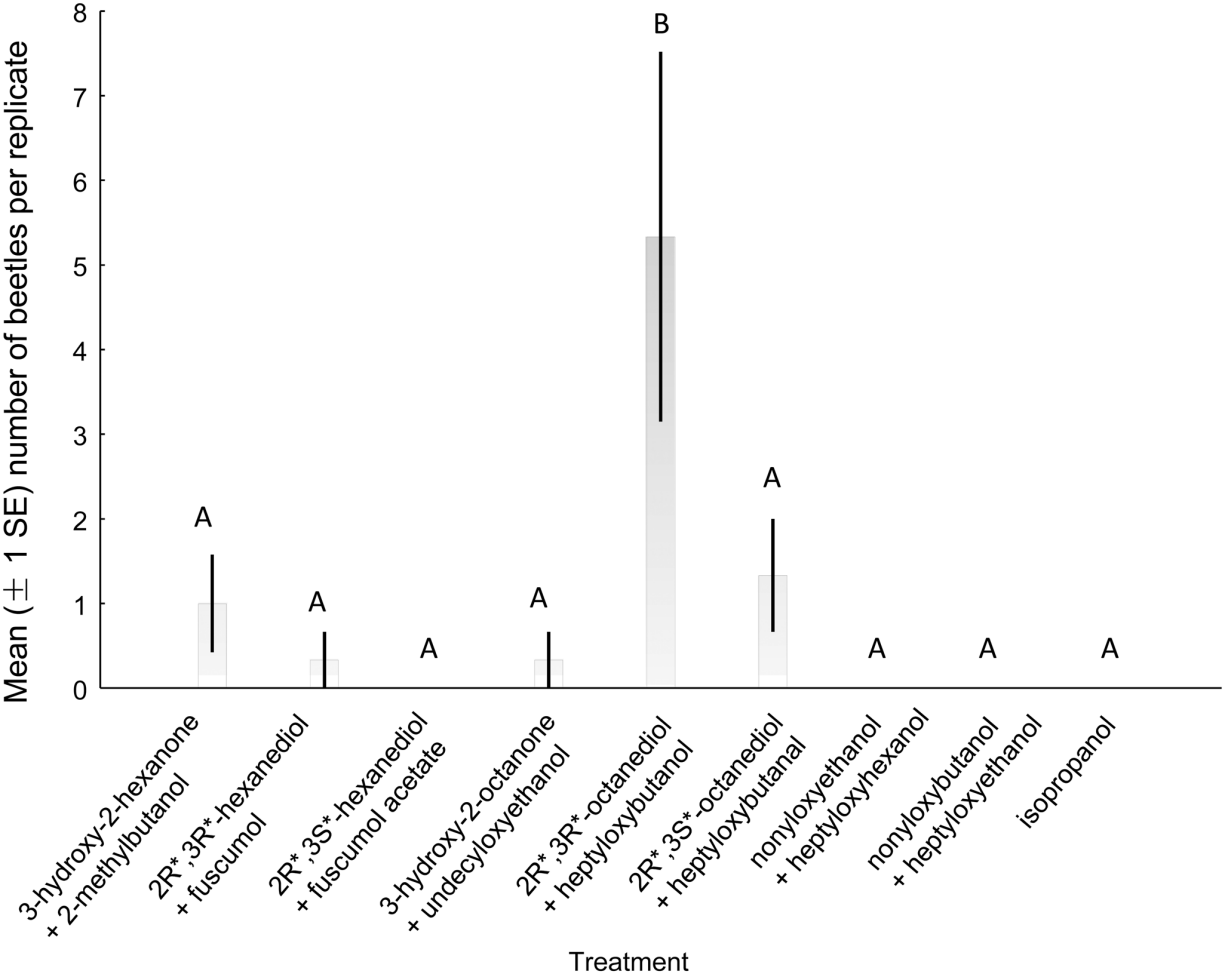
Mean (± 1 SE) numbers of adult male and female *Anoplophora chinensis* captured by traps baited with synthetic pheromone in Nanjing, China. Chemical abbreviations: alcohol, 4-(n-heptyloxy)butan-1-ol; aldehyde, 4-(n-heptyloxy)butanal. Within each sex, different letters indicate significant differences between pairs of treatments (Duncan’s range test, *P* > 0.05).

A total of 97 adult *A*. *chinensis* (42 females, 55 males) were captured during field experiments at the two study sites. Because there was no significant lure x site interaction (male and female trap captures combined and log[x+1] transformed; ANOVA F_3,52_ = 0.316, p = 0.81), trap captures from the two sites were analyzed together. Traps baited with the alcohol alone or in a 1:1 blend with the aldehyde caught significantly more beetles of both sexes than those baited with aldehyde alone, or control traps (Fig 4; males, ANOVA *F*_3,56_ = 5.24, *P* < 0.005, females, Kruskal-Wallis *H*_3,60_ = 22.0, *P* < 0.0001). Traps baited with the two attractive treatments (i.e., alcohol alone and the blend) captured males and females in approximately equal numbers (χ^2^ = 0.023, *P* = 0.88; χ^2^ = 0.61, *P* = 0.43, respectively).

## Discussion

Attraction of adult *A*. *chinensis* to 4-(n-heptyloxy)butan-1-ol, alone or in a blend with the aldehyde, provides strong evidence that the alcohol is an important component of the volatile pheromone produced by males. Furthermore, the fact that beetles of both sexes were attracted indicated that the alcohol is an aggregation pheromone according to the formal definition (but see [22]), even though its primary function is likely to be bringing the sexes together for mating.

Low overall trap catches in our field trials may have been due to several factors. First, at the Nanjing Botanical Garden field site, we did not observe any adults outside of traps, suggesting that the beetle population at this site was low. Second, because these trials were intended only to provide evidence of a male-produced pheromone, only a single pheromone release rate was tested. Thus, we expect that optimization of the release rate in follow-up studies will result in more effective lures. Third, it is possible that the pheromone may be strongly synergized by host plant volatiles, as has been found for a number of other species in the same tribe [23,24]. Indeed, we observed antennal responses to host volatiles (Fig 3).

The fact that *A*. *chinensis* and *A*. *glabripennis* share the alcohol as a pheromone component is analogous to the trends seen within other closely related cerambycids, even for congeners on different continents that presumably have been separated for millions of years [19,25,26]. Because the two *Anoplophora* species are sympatric over much of their ranges, and have many host species in common [1], it is unclear how cross-attraction is avoided, given their mutual attraction to the alcohol as a single component, or blended with the aldehyde [10,18,27–28]. However, recent studies have shown that sympatric cerambycid species that share pheromone components may avoid cross-attraction by using alternative reproductive isolation strategies, such as adults having different seasonal and/or diel activity periods [29]. The two *Anoplophora* species may use similar strategies. Alternatively, there also may be subtle differences between the two species in host utilization strategies, as suggested by data from quarantine interceptions: *A*. *glabripennis* has almost always been intercepted in wooden packing materials (i.e., boards cut from felled trees; 96% of interceptions), whereas *A*. *chinensis* has been almost invariably found in living woody plants that have been imported (99% of interceptions; [2]). However, on multiple occasions we have observed adults of both species on the same host trees in Nanjing, China. In such cases, cross breeding probably is inhibited by the species specificity of contact pheromones in the cuticular lipids, as is true for other species of cerambycid beetles [30].

In summary, the data described above suggest that 4-(*n*-heptyloxy)butanol would be an excellent candidate for a lure for monitoring invasive populations of *A*. *chinensis* in Europe, and for detecting new invasions in North America and other parts of the world.

## Supporting Information

S1 Fig Trap captures of *Anoplophora chinensis* adults in traps baited with cerambycid pheromones in Nanning, China in 2011. A preliminary experiment assessing the attraction of *A*. *chinensis* to 4-(*n*-heptyloxy)butan-1-ol and 4-(*n*-heptyloxy)butan-1-al was conducted as part of broader screening trials in China. The experiment was conducted at Qingxiushan Park, Nanning, Guangxi (22°47'6.00"N, 108°22'53.52"E) during 13 April—12 May 2011, using identical compounds, formulations, lure devices, and traps as previously described [17], but with four additional treatments: 25 mg of 4-(*n*-heptyloxy)butan-1-ol, 25 mg of 4-(*n*-heptyloxy)butanal, 10 mg (*Z*)-3-decenyl (*E*)-2-hexenoate, and 50 mg racemic 2-methylbutan-1-ol, each diluted to 1 ml with ethanol and tested separately. There were two spatial replicates and traps were rotated one position on 28 April (N = 4). Trap captures were analyzed by Kruskall-Wallis ANOVA and Median test. A total of 20 adult *A*. *chinensis* were captured including 16 in traps baited with 4-(*n*-heptyloxy)butan-1-ol, and 4 beetles in the 4-(*n*-heptyloxy)butanal traps. No *A*. *chinensis* of either sex were captured in other treatments or controls. There was a significant treatment effect (Kruskal-Wallis ANOVA H_15,64_ = 29.4, *P* = 0.0143) and the median test showed that significantly more *A*. *chinensis* adults were attracted to 4-(*n*-heptyloxy)butan-1-ol compared to blank controls (S1 Fig, Median test χ^2^_15,64_ = 29.9, *P* = 0.0124) with a significant male bias (1:2 F:M; χ^2^ = 4.5, *P* < 0.05).
(TIF)

S2 Fig Trap captures of *Anoplophora chinensis* adults in traps baited with binary combinations of cerambycid pheromone candidates in Nanjing, China in 2012. A second preliminary experiment was conducted at the Nanjing Botanical Garden (site location and experimental methods as described in the methods section) during 30 May– 22 July 2012. The treatments were the following binary combinations plus an isopropanol control: (1) racemic 3-hydroxy-2-hexanone + racemic 2-methylbutan-1-ol, (2) (2R*,3R*)-hexanediol + racemic fuscumol, (3) (2R*,3S*)-hexanediol + racemic fuscumol acetate, (4) 3-hydroxy-2-octanone + monochamol, (5) (2R*,3R*)-octanediol + 4-(*n*-heptyloxy)butan-1-ol, (6) (2R*,3S*)-octanediol + 4-(*n*-heptyloxy)butanal, (7) nonyloxyethanol + heptyloxyhexanol, (8) nonyloxybutanol + heptyloxyethanol. All racemic compounds were used at a dose of 50 mg/lure, whereas individual pure compounds were loaded at 25 mg/lure. There were three spatial replicates. Because raw trap catches did not meet the assumption of homogeneity of variances, the data were transformed (log[x+1]) and subjected to ANOVA followed by Duncan’s range test. The treatment effect was significant (F_8,21_ = 5.8, P<0.001) and only the treatment that included 4-(*n*-heptyloxy)butan-1-ol trapped significantly more *A*. *chinensis* adults than the controls (P<0.05).
(TIF)
