# Correction: Identification of a Male-Produced Pheromone Component of the Citrus Longhorned Beetle, *Anoplophora chinensis*

**DOI:** 10.1371/journal.pone.0145355

**Published:** 2015-12-16

**Authors:** Laura Hansen, Tian Xu, Jacob Wickham, Yi Chen, Dejun Hao, Lawrence M. Hanks, Jocelyn G. Millar, Stephen A. Teale

Fig 4, “Mean (± 1 SE) numbers of adult male and female *Anoplophora chinensis* captured by traps baited with synthetic pheromone in Nanjing, China,” is a duplicate of S2 Fig. Please view the correct Fig 4 here.

**Fig 4:**
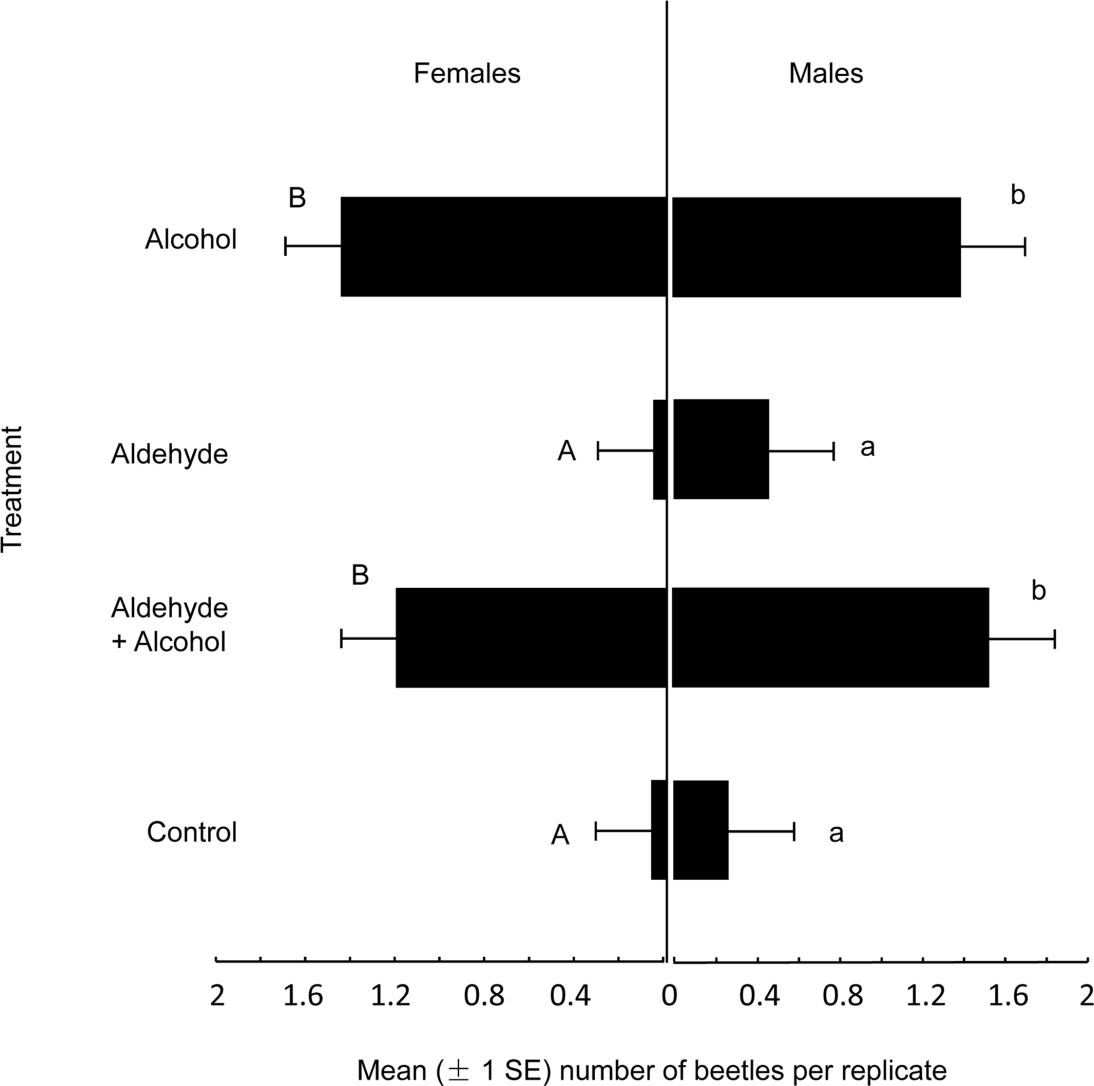
Mean (± 1 SE) numbers of adult male and female *Anoplophora chinensis* captured by traps baited with synthetic pheromone in Nanjing, China. Chemical abbreviations: alcohol, 4-(n-heptyloxy)butan-1-ol; aldehyde, 4-(n-heptyloxy)butanal. Within each sex, different letters indicate significant differences between pairs of treatments (Duncan’s range test, *P* > 0.05).
