# Supplementary figures and images for: Identification of a Male-Produced Pheromone Component of the Citrus Longhorned Beetle, Anoplophora chinensis
Source: PLoS One. 2015 Aug 4;10(8):e0134358. doi: 10.1371/journal.pone.0134358 (PMC4524714; doi:10.1371/journal.pone.0134358)

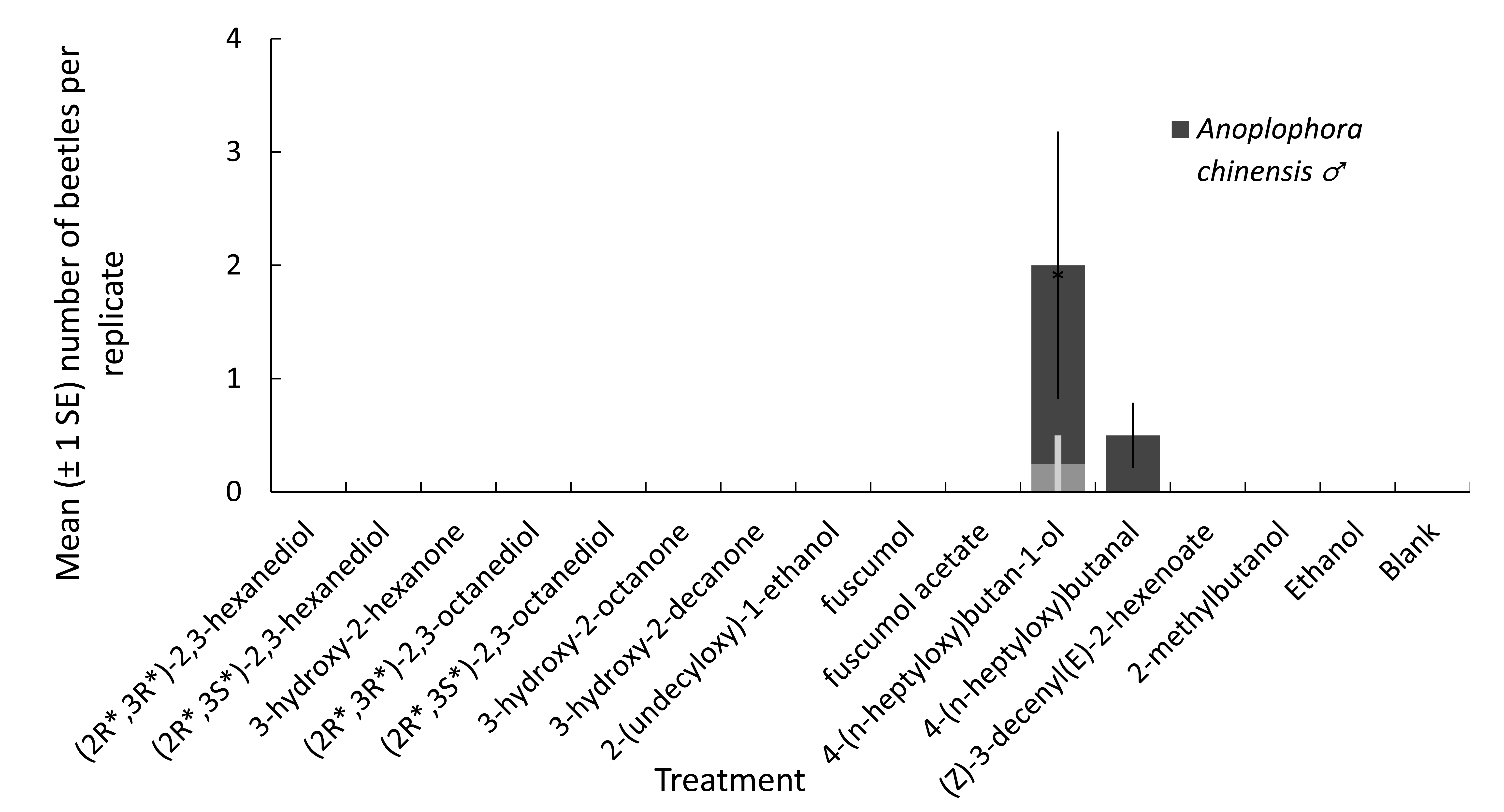

Supplement: S1 Fig — A preliminary experiment assessing the attraction of A. chinensis to 4-(n-heptyloxy)butan-1-ol and 4-(n-heptyloxy)butan-1-al was conducted as part of broader screening trials in China. The experiment was conducted at Qingxiushan Park, Nanning, Guangxi (22°47'6.00"N, 108°22'53.52"E) during 13 April—12 May 2011, using identical compounds, formulations, lure devices, and traps as previously described [17], but with four additional treatments: 25 mg of 4-(n-heptyloxy)butan-1-ol, 25 mg of 4-(n-heptyloxy)butanal, 10 mg (Z)-3-decenyl (E)-2-hexenoate, and 50 mg racemic 2-methylbutan-1-ol, each diluted to 1 ml with ethanol and tested separately. There were two spatial replicates and traps were rotated one position on 28 April (N = 4). Trap captures were analyzed by Kruskall-Wallis ANOVA and Median test. A total of 20 adult A. chinensis were captured including 16 in traps baited with 4-(n-heptyloxy)butan-1-ol, and 4 beetles in the 4-(n-heptyloxy)butanal traps. No A. chinensis of either sex were captured in other treatments or controls. There was a significant treatment effect (Kruskal-Wallis ANOVA H15,64 = 29.4, P = 0.0143) and the median test showed that significantly more A. chinensis adults were attracted to 4-(n-heptyloxy)butan-1-ol compared to blank controls (S1 Fig, Median test χ2 15,64 = 29.9, P = 0.0124) with a significant male bias (1:2 F:M; χ2 = 4.5, P < 0.05). (TIF) [file pone.0134358.s001.tif]

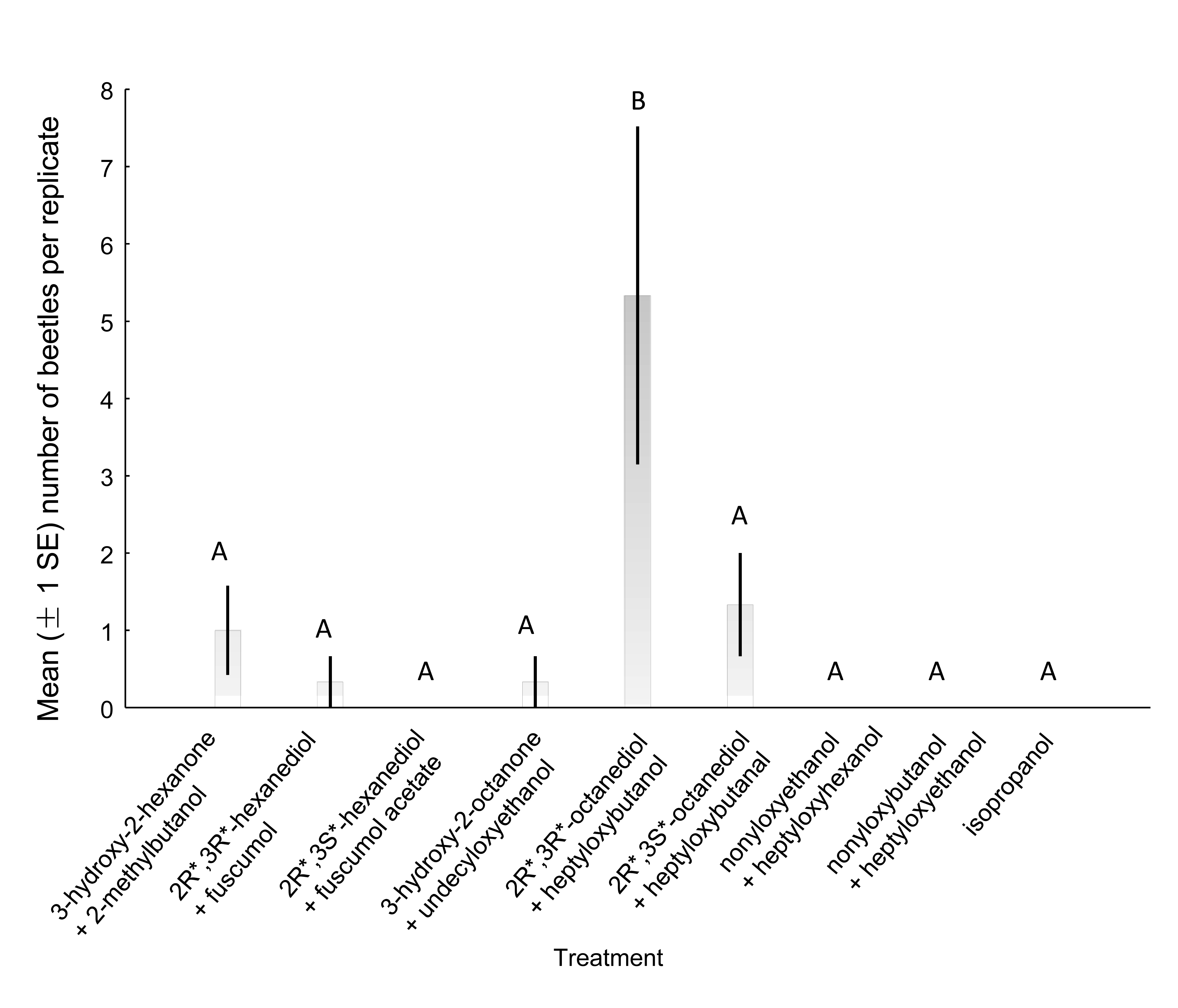

Supplement: S2 Fig — A second preliminary experiment was conducted at the Nanjing Botanical Garden (site location and experimental methods as described in the methods section) during 30 May– 22 July 2012. The treatments were the following binary combinations plus an isopropanol control: (1) racemic 3-hydroxy-2-hexanone + racemic 2-methylbutan-1-ol, (2) (2R*,3R*)-hexanediol + racemic fuscumol, (3) (2R*,3S*)-hexanediol + racemic fuscumol acetate, (4) 3-hydroxy-2-octanone + monochamol, (5) (2R*,3R*)-octanediol + 4-(n-heptyloxy)butan-1-ol, (6) (2R*,3S*)-octanediol + 4-(n-heptyloxy)butanal, (7) nonyloxyethanol + heptyloxyhexanol, (8) nonyloxybutanol + heptyloxyethanol. All racemic compounds were used at a dose of 50 mg/lure, whereas individual pure compounds were loaded at 25 mg/lure. There were three spatial replicates. Because raw trap catches did not meet the assumption of homogeneity of variances, the data were transformed (log[x+1]) and subjected to ANOVA followed by Duncan’s range test. The treatment effect was significant (F8,21 = 5.8, P<0.001) and only the treatment that included 4-(n-heptyloxy)butan-1-ol trapped significantly more A. chinensis adults than the controls (P<0.05). (TIF) [file pone.0134358.s002.tif]
